# Aberrant prefrontal functional connectivity during verbal fluency test is associated with reading comprehension deficits in autism spectrum disorder: An fNIRS study

**DOI:** 10.3389/fpsyg.2022.984777

**Published:** 2022-09-20

**Authors:** Melody M. Y. Chan, Ming-Chung Chan, Michael K. Yeung, Shu-Mei Wang, Duo Liu, Yvonne M. Y. Han

**Affiliations:** ^1^Department of Rehabilitation Sciences, The Hong Kong Polytechnic University, Kowloon, Hong Kong SAR, China; ^2^Department of Psychology, The Education University of Hong Kong, Hong Kong, Hong Kong SAR, China; ^3^Department of Special Education and Counselling, The Education University of Hong Kong, Hong Kong, Hong Kong SAR, China; ^4^University Research Facility in Behavioral and Systems Neuroscience (UBSN), The Hong Kong Polytechnic University, Kowloon, Hong Kong SAR, China

**Keywords:** autism, reading comprehension, executive functioning, verbal fluency, processing speed, functional connectivity, fNIRS

## Abstract

Children with autism spectrum disorder (ASD) show marked difficulties in reading comprehension, a complex cognitive skill fundamental to successful daily functioning that is associated with core executive functions. However, the neurophysiological mechanisms underlying reading comprehension deficits in these children remain elusive. Twenty-one right-handed males with high-functioning ASD (mean age = 10.24 years) and 23 age-, IQ-, educational level-, sex- and handedness-matched typically developing (TD; mean age = 10.14 years) individuals underwent a reading comprehension test and the semantic verbal fluency test that tapped core executive functions underlying reading comprehension during concurrent prefrontal functional near-infrared spectroscopy (fNIRS) measurement. Participants’ information processing efficiency was also assessed. High-functioning ASD children exhibited general reading comprehension [main effect of group: *F*_(1,40)_ = 7.58, *p* = 0.009], selective verbal fluency deficits [Group × category interaction: *F*_(1,42)_ = 4.90, *p* = 0.032] and slower processing speed (*t*_42_ = 2.36, *p* = 0.023). Regarding the hemodynamics of the prefrontal cortex (PFC), although ASD individuals showed comparable patterns of PFC brain activation to their healthy counterparts, lower PFC intrahemispheric [main effect of group: *F*_(1,42)_ = 11.36, *p* = 0.002] and interhemispheric [main effect of group: *F*_(1,42)_ = 7.79, *p* = 0.008] functional connectivity were evident during the semantic verbal fluency test. At the whole-group level, poorer reading comprehension performance was associated with poorer performance in the semantic verbal fluency test (*r*_42_ = 0.508, *p* < 0.001). Moreover, poorer semantic verbal fluency test performance was associated with slower information processing speed (*r*_42_ = –0.312, *p* = 0.044), which is associated with reduced left medial PFC functional connectivity (*r*_42_ = –0.319, *p* = 0.040). Abnormal intrahemispheric and interhemispheric prefrontal hypoconnectivity is associated with deficits in executive processes essential for reading comprehension in ASD. Our study has provided important implications for the neuropsychological and neurophysiological mechanisms underlying reading comprehension deficits in ASD.

## Introduction

Autism spectrum disorder (ASD) is a pervasive neurodevelopmental disorder characterized by language and communication deficits as well as repetitive, stereotyped behaviors (American Psychological Association, 2013). Previous research has shown that individuals with ASD encounter great difficulties in reading comprehension (Jones et al., 2009; Brown et al., 2013), which is a fundamental skill for knowledge acquisition and communicating with others in daily life (Pretorius, 2002; Moje et al., 2011; Woolley, 2011) that predicts academic success (Vilenius-Tuohimaa et al., 2008; García-Madruga et al., 2014). According to the Simple View of Reading (Gough and Tunmer, 1986; Hoover and Gough, 1990), reading comprehension involves the processes of word recognition (i.e., decoding) and the interpretation of lexical information, sentences and discourses. Notably, these processes are heavily mediated by the knowledge of vocabulary (i.e., understanding of the semantic meaning of a single word; Hoover and Gough, 1990; Cromley and Azevedo, 2007), with a greater vocabulary size usually being associated with better vocabulary knowledge (Dong et al., 2020). Although ASD individuals have been shown to possess intact abilities in word recognition (Venker et al., 2021) and the phonetic decoding of non-words (Gabig, 2010), they are found to exhibit consistent deficits in the interpretation of linguistic components when they read (Kim et al., 2014). In addition, previous neuropsychological studies have suggested that reading involves the integration and execution of different cognitive-perceptual processes (Christopher et al., 2012; Kendeou et al., 2016). For example, when comprehending a written sentence, the individual words are first recognized, and then temporarily stored in the brain for matching with the learned phonological, orthographic, and semantic representations, followed by combining these representations to form an understanding of the sentence (Christopher et al., 2012). For the successful comprehension of a longer sentence or even a passage with multiple paragraphs conveying several ideas, a reader must process more information that is relevant to a given question, and maintain the new information while disengaging from the old information (Hansen et al., 2017; Martin et al., 2020). In other words, the demand for cognitive-perceptual processes increases when the comprehension task becomes more complex. In the case of ASD, a previous study has shown that the complexity of questions plays a role in mediating the reading comprehension performance in these individuals (Tirado and Saldana, 2016).

Previous studies have shown that many core components of executive functions, namely, updating, inhibition and shifting (Miyake et al., 2000), are involved in reading comprehension. For instance, a review conducted by Butterfuss and Kendeou (2018) concluded that updating supports comprehension by maintaining the relevant information in the working memory system, inhibition supports comprehension through the suppression of irrelevant text information and prevention of irrelevant thoughts from intruding the working memory system, and shifting aids attention allocation to different features of the text. Importantly, an accumulative body of evidence has established a clear relationship between these executive processes and reading comprehension (Carretti et al., 2005, 2009; Savage et al., 2006; Kim, 2020), and deficits in executive functions have been shown to be associated with poor reading comprehension performance in less competent readers (Swanson et al., 2006). Because many previous studies have revealed executive dysfunctions in ASD (Demetriou et al., 2018), impairments in executive function in ASD may be associated with reading comprehension difficulties in these individuals. Indeed, a previous study has shown that individuals with ASD are less efficient in reading written text, with text reading time being significantly associated with the performance of an executive function task involving planning and visuospatial working memory (Micai et al., 2021).

Among many executive function tasks, the semantic verbal fluency (VF) task involves the interplay between executive function and language process. For this test, a task-taker is required to voluntarily generate as many words as possible with a given semantic category and avoid giving repetitive and irrelevant answers (Lezak et al., 2004). In other words, this task gives a direct quantitative measure of the vocabulary size of an individual. As discussed above, given vocabulary knowledge is fundamental to reading comprehension processes and vocabulary size can be one of the key parameters reflecting one’s level of vocabulary knowledge, the semantic VF task is a suitable task for generating insights into the executive processes underlying reading comprehension. Evidence from neuroimaging meta-analyses has shown that the semantic VF task activates brain networks that primarily involve the frontal lobe. While left medial, superior and inferior frontal gyri are found to be activated for VF tasks performed in non-tonal language (e.g., English, Wagner et al., 2014), bilateral activations involving superior, middle and inferior frontal gyri are found when VF tasks are performed in tonal language (i.e., Chinese; Wu et al., 2012). Evidence from functional connectivity studies shows that the semantic VF task involves prefrontal functional connectivity changes. Specifically, a left-lateralized increase in connections accompanied by an increase in cerebral blood flow at superior (BA6), middle (BA6) and inferior (BA9,45,47) frontal gyri has been shown during the semantic VF task when compared to baseline (Paschoal et al., 2021). Multiple lines of evidence from lesion studies show that functional segregation for the medial and lateral prefrontal cortex (PFC) is evident for semantic VF. For instance, while the lateral PFC has been shown to be associated with the strategic retrieval of words (Reverberi et al., 2006), the superior and medial prefrontal cortices have been shown to involve the voluntary generation of new words relevant to the given cues (Robinson et al., 2012).

Some previous studies have provided evidence to support semantic VF impairment in ASD. Specifically, Inokuchi and Kamio (2013) and Yeung et al. (2019) have shown that individuals with ASD showed significantly poorer performance than typically developing (TD) individuals only for living (i.e., animal) but not nonliving (i.e., transport) categories, suggesting a category-dependent impairment in semantic VF in ASD. However, Ehlen et al. (2020) showed that ASD individuals have intact ability in living-category semantic VF. Similar conflicting results are documented in neurophysiological and neuroimaging studies. For instance, a previous functional magnetic resonance imaging (fMRI) study showed that ASD individuals exhibited reduced activation in the left inferior and middle frontal gyri (Kenworthy et al., 2013); conversely, another fMRI study showed enhanced activation in the left inferior frontal gyrus during a similar semantic VF task (Beacher et al., 2012). For functional near-infrared spectroscopy (fNIRS) studies, although Yeung et al. (2019) showed that ASD individuals exhibited a significantly altered activation pattern in the medial and lateral PFC when compared to TD individuals, Ota et al. (2020) revealed non-significant differences between ASD and TD individuals in prefrontal activation when a similar fNIRS paradigm was used.

Some researchers have suggested that semantic VF task performance in ASD may be mediated by information processing speed (Carmo et al., 2015), i.e., the time needed for information travel across the brain indexed by a reaction time during task performance (Neubauer and Fink, 2003). Because ASD has long been construed as an information processing disorder (Minshew et al., 2008), indicated by an increase in reaction time during various executive function tasks (Chan et al., 2022), slow processing speed might be associated with semantic VF performance. A number of studies have shown that slow processing speed is associated with abnormal structural and functional connectivity (Silva et al., 2020), and previous studies have shown that functional connectivity is altered in ASD during resting-state (Han and Chan, 2017; King et al., 2019) and cognitive task performance (e.g., working memory, Koshino et al., 2005).

As reviewed above, given semantic VF task (1) is an executive function task involving language processes that is sensitive in tapping cognitive processes fundamental to reading comprehension, (2) requires intact functioning of the medial and lateral PFC, and (3) is influenced by information processing efficiency contributed by functional connectivity in the brain altered functional connectivity in the medial and/or lateral PFC during semantic VF task could be the possible neurophysiological mechanism associated with reading comprehension deficits in ASD. However, to the best of our knowledge, the prefrontal functional connectivity of ASD during semantic VF tasks and its behavioral correlates have not been investigated in previous studies. Specifically, abnormalities in both intrahemispheric and interhemispheric functional connectivity have been reported in previous resting-state fMRI and fNIRS studies in ASD (Anderson et al., 2011; Zhu et al., 2014; Lee et al., 2016; Guo et al., 2020), but these changes have not yet been examined when these individuals perform the VF task. This study aimed to investigate the neurophysiological mechanism underlying reading comprehension performance in children with high-functioning ASD. Compared to typically developing (TD) individuals, ASD individuals were hypothesized to show impaired reading comprehension performance, especially when task difficulty increases, which is associated with impaired semantic VF performance. In turn, impaired semantic VF performance in ASD is associated with reduced information processing efficiency, which is associated with altered prefrontal functional connectivity and activation patterns.

## Materials and methods

### Participants

This study was approved by the Human Subjects Ethics Sub-Committee of the Hong Kong Polytechnic University and was conducted in accordance with the Declaration of Helsinki. Twenty-one individuals with ASD and 23 TD individuals aged 8–12 years who were studying for grades 3–5 at local mainstream primary schools participated in this study. All participants achieved a full intelligence quotient (IQ) of ≥ 80 measured by the short form of the Hong Kong Wechsler Intelligence Scale for Children—fourth edition (WISC-IV-HK:SF, Wechsler, 2010). Because previous research has shown that ASD individuals exhibit sex-dependent differences in nonsocial cognitive domains involving executive function (Lai et al., 2012), we only included males in our sample. Furthermore, as prior studies have also shown that handedness influenced the neural connectivity underlying Chinese semantic language processing (Gao et al., 2015), we only included right-handed individuals whose handedness was confirmed by scoring over +80 on the Edinburgh Handedness Inventory short form (Oldfield, 1971). The diagnosis of ASD in participants was confirmed with the Autism Diagnostic Interview—Revised (ADI-R, Lord et al., 1994), which was conducted by a clinical psychologist blinded to the hypothesis of the study. The social functioning of TD individuals was screened with the second edition of the Social Responsiveness Scale (SRS-2, Constantino and Gruber, 2012). All included TD individuals obtained a T-score equal to or below 59, indicating that they had normal daily social functioning.

### Procedure and materials

Before the commencement of the experiment, the procedures and potential risks and benefits of the study were first explained to the participants and their parents, and written informed consent was then obtained from all participating parents of the recruited individuals. All children underwent a three-part assessment, including a WISC-IV-HK:SF IQ assessment, a series of behavioral assessments and fNIRS measurements. The sequence of assessments was counterbalanced across subjects to minimize order effects. While parents of all children were asked to complete the SRS-2, parents of children with ASD were involved in a structured interview with ADI-R administered in addition to the completion of the SRS-2. The WISC-IV-HK:SF and ADI-R were administered by a clinical psychologist. The behavioral assessments and fNIRS measurements were conducted by trained research assistants.

#### Behavioral assessments

Behavioral assessments were conducted to examine the reading comprehension ability and information processing efficiency of the participants.

The reading comprehension test consisted of three parts with increasing difficulty. The first part, which consisted of three short sentences of 20–35 words, assessed children’s pronoun resolution ability. The second part consisted of a 50-word short descriptive paragraph, from which participants were required to answer two questions asking for the identification of specific details from the paragraph. The third part consisted of two passages with multiple paragraphs. The first passage was the story behind a Chinese idiom with conversations between two people embedded in the story. Children were asked not only to identify specific details from the passage but also to make logical conclusions and infer the symbolic meanings of a word and the whole passage. The second passage was a 200-word metaphorical description of the Great Wall of China, with all six questions testing the participants’ understanding of the metaphors used in the passage. No time limit was enforced for test completion. A higher score obtained on the test indicated better reading comprehension performance.

The participants’ information processing efficiency was assessed using the five-choice reaction time (RTI) test from the *Cambridge Neuropsychological Test Automated Battery* (CANTAB^®^, Fray et al., 1996). This test is a standardized computerized test tapping participants’ mental processing speeds by eliminating the effect of movement execution time (Sachse et al., 2013). Five-choice mean reaction time, a commonly adopted measure for reflecting information processing efficiency (Amir et al., 2020), was used. A slower mean reaction time indicates less efficient information processing.

#### Functional near-infrared spectroscopy measurement

**FIGURE 1:**
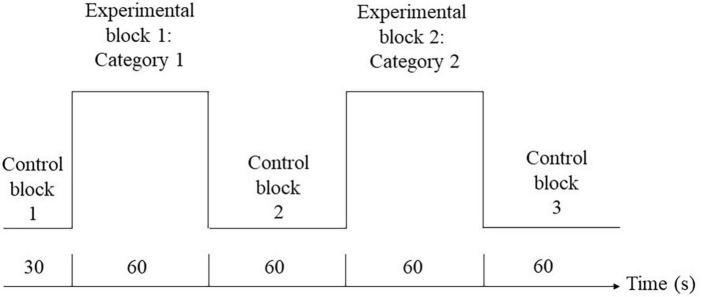
Semantic verbal fluency task design.

**FIGURE 2:**
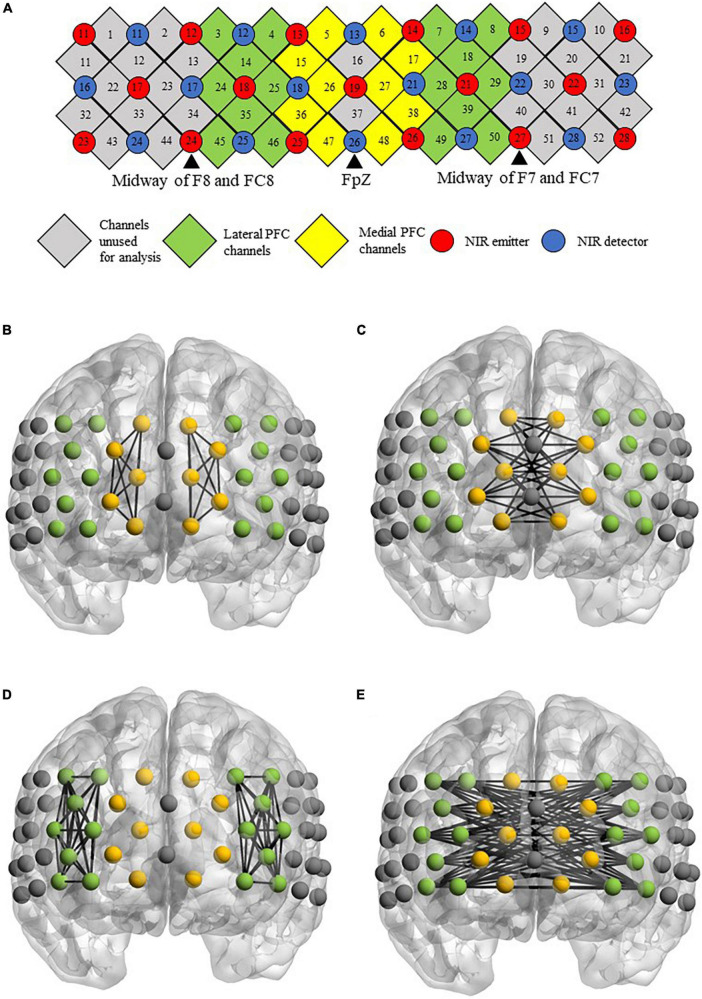
**(A)** Arrangements of channels, near-infrared (NIR) emitters, detectors and channel groupings. **(B–E)** Figures showing the location of channels on a brain template. The figures define the functional connectivity of the **(B)** intrahemispheric and **(C)** interhemispheric medial PFC, as well as the functional connectivity of the **(D)** intrahemispheric and **(E)** interhemispheric lateral PFC.

fNIRS measurements were taken for each of the participants when they performed the semantic verbal fluency task. The paradigm was adapted from a previous study (Yeung et al., 2019), and the task design is illustrated in Figure 1. During the experimental blocks, a category word (i.e., “animal” or “transport”) was shown in the center of the screen. Participants were instructed to orally produce as many words relevant to the given category as possible without repeating their ideas in 60 s. The order of the experimental blocks was counterbalanced across individuals. During the control blocks, participants were instructed to repeat the phrase “1, 2, 3, 4” slowly and continuously in the given period of time (i.e., 30 s for the first control block, 60 s for the second and third control blocks). The visual stimuli were presented using E-prime 3 (Psychology Software Tools Inc., Sharpsburg, PA, USA). Changes in hemodynamics in the prefrontal cortex during the VF task were measured by a 52-channel fNIRS optical topography system (ETG-4000; Hitachi Medical Co., Tokyo, Japan). The NIR probe was set with thirty-three emitters and detectors mounted on a 3 × 11 grid and was worn on a participant’s head using an elastic headband, with detector 26 placed at FpZ, emitter 24 placed midway between F8 and FC8, and emitter 27 placed midway between F7 and FC7 for children aged 8–11 years old with an average head circumference of 52 cm in our sample. The distance between each emitter and detector was fixed at 30 mm. The channels were grouped to generate the medial and lateral PFC regions of interest in the left and right hemispheres (Yeung et al., 2019). The NIRS probe set and channel groupings are shown in Figure 2.

### Data analysis

#### Functional near-infrared spectroscopy preprocessing

fNIRS data preprocessing was performed using the AnalyzIR toolbox (Santosa et al., 2018) in MATLAB 2019a (The Mathworks, Natick, MA). The preprocessing pipeline was reported elsewhere (Chan et al., 2022; Han et al., 2022). First, bad channels were interpolated using the *FixSatChans* or *FixFlatChans* modules available in the toolbox. To filter the cardiac signals that contribute to the serial autocorrelations commonly observed in fNIRS data, the light intensity data were downsampled to 1 Hz. To remove excessive baseline before and after the task, the *TrimBaseline* module was applied to keep the first control block as the baseline only. The interpolated, downsampled and trimmed data were then converted to optical density, followed by conversion to oxyhaemoglobin (HbO), deoxyhaemoglobin (HbR), and total hemoglobin (HbT) concentrations using the modified Beer–Lambert Law. HbO data were analyzed in this study because this parameter has been shown to be more sensitive in detecting task-related neural changes in patients with neuropsychiatric disorders (Yeung and Lin, 2021).

#### Analysis of individual functional near-infrared spectroscopy data

Both prefrontal fNIRS activation and functional connectivity data were analyzed at the individual level. First, the differences in prefrontal activation between the experimental (i.e., animal/transport word generation) and baseline (i.e., number repetition) conditions were estimated with a general linear model with an autoregressive prewhitening filter using iteratively reweighted least-squares (AR-IRLS). The method is described in detail in Barker et al. (2013). In brief, for each fNIRS channel, the AR-IRLS algorithm first calculates a regression coefficient using the ordinary least square (OLS) estimator. The residual from the OLS model is then fit to a fifth-order autoregressive (AR) model because a previous simulation study has shown that a fifth-order model is sufficient to remove serially correlated signals without overfitting (Barker et al., 2013). By using the Cochrane-Orcutt method, the AR model coefficient is further used to design a whitening filter that removes the correlations in the signal between consecutive time points. Given that a linear model of the evoked response in fNIRS data can be represented by *y* = *X*β+ε (Penny et al., 2011), the whitening filter is applied to both the design matrix (*X*) and the fNIRS (*y*), yielding a denoised signal for iteratively reweighted least squares (IRLS) and finally solving the IRLS to obtain the channelwise beta value (β) for subsequent analysis. Regarding functional connectivity analysis, for each of the possible channel pairs, the correlation between the β-values of two individual channels was estimated by a robust regression approach (Shevlyakov and Smirnov, 2011; Santosa et al., 2017), yielding a correlation coefficient (R), which was then Z-transformed (Z). The β- and Z values were averaged within each region of interest (i.e., left medial PFC, right medial PFC, left lateral PFC and right lateral PFC) for both animal and transport conditions (Figure 2). These values were used in the second-level fNIRS activation and functional connectivity analysis.

#### Group-level data analysis

Group-level analyses of behavioral and fNIRS data were performed using IBM SPSS Statistics Version 26.0 (IBM Corp., Armonk, NY). The normality of the data was first checked with the Shapiro–Wilk test. For non-normal behavioral and fNIRS data, the Aligned Rank Transform Contrasts [ART-C, (Elkin et al., 2021)] procedure was carried out to facilitate the administration of omnibus tests. Extreme outliers (i.e., values larger than the 75-percentile + 3* interquartile range/values smaller than the 25-percentile – 3* interquartile range) were removed from subsequent analyses.

To examine whether the TD and ASD groups were matched, independent sample *t*-tests (or Mann–Whitney tests for non-normal data) were performed for age, IQ, educational level and SRS-2 data. To test for the difference in reading comprehension performance between the two groups across different levels of difficulty (H_1_), subscores (from different levels) from the reading comprehension test were analyzed with 2 *(group)* × 3 *(level of difficulty)* mixed ANOVA. To test for the difference in semantic verbal fluency performance between the two groups across different word categories (H_2_), the total numbers of correct words generated for each category (i.e., animal or transport) were analyzed with 2 (group) × 2 (category) mixed ANOVA. To test for the difference in information processing efficiency between the two groups (H_3_), an independent sample *t*-test (or Mann–Whitney test for non-normal data) was performed for the five-choice mean reaction time from the CANTAB reaction time test. To investigate the difference in prefrontal fNIRS activation patterns between the two groups during different categories in the semantic verbal fluency test in the medial/lateral PFC of the left/right hemisphere (H_4_), a 2 (group) × 2 (category) × 2 (region) × 2 (hemisphere) mixed ANOVA was performed with the averaged β-values of the medial/lateral PFC of each hemisphere. To investigate the difference in prefrontal fNIRS functional connectivity between the two groups during different categories in the semantic verbal fluency test within the medial/lateral PFC of the left/right hemisphere (H_5_), a 2 (group) × 2 (category) × 2 (region) × 2 (hemisphere) mixed ANOVA was performed with the averaged Z values of the medial/lateral PFC of each hemisphere. For the above analyses, we focused on the interaction effect with group as well as the main effect of group, as this study intended to investigate the group difference between ASD and TD. Bonferroni corrections were applied to each of the hypotheses to correct for multiple comparisons. To explore the relationship between brain hemodynamic changes and behavioral performance, Pearson’s correlation analyses were performed at both the whole-group and subgroup levels for parameters found significant in the group comparisons. While the significance level for these exploratory analyses was kept at *p* = 0.05 (uncorrected), we also reported results with a *p*-value smaller than 0.1 given the exploratory nature of these analyses.

## Results

### Demographic details

The demographic details of the participants are listed in Table 1. Independent sample *t*-tests revealed that the ASD and TD groups were matched in age, full-scale IQ and educational level (*p*s > 0.158). Our ASD sample also showed markedly impaired current social functioning when compared to their TD counterparts, indicated by a statistically higher SRS-2 total score (*p* < 0.001).

**TABLE 1:** Participants’ demographic information.

Parameters	Group				
	ASD	TD	*t*	*df*	*p*
Mean chronological age in years (*S.D.*)	10.24 (1.16)	10.14 (0.90)	0.339	42	0.736
Mean full scale IQ (*S.D.*)	100.76 (16.04)	105.74 (12.11)	1.17	42	0.249
Educational level (*S.D.*)	3.90 (1.00)	4.30 (0.82)	1.46	42	0.153
Mean SRS-2 total score (*S.D.*)	84.00 (22.32)	42.89 (14.92)	6.64***	42	<0.001
**Mean ADI-R domain scores (*S.D.*)**					
Social interaction	14.26 (7.58)	N/A	N/A		
Communication	10.84 (5.87)				
Restricted, repetitive behavior	4.63 (2.17)				

All participants (ASD *n* = 21, TD *n* = 23) were right-handed male.

SRS, Social Responsiveness Scale- 2; ADI-R, Autism Diagnostic Interview- Revised.

***p < 0.001.

### Behavioral performance

**FIGURE 3:**
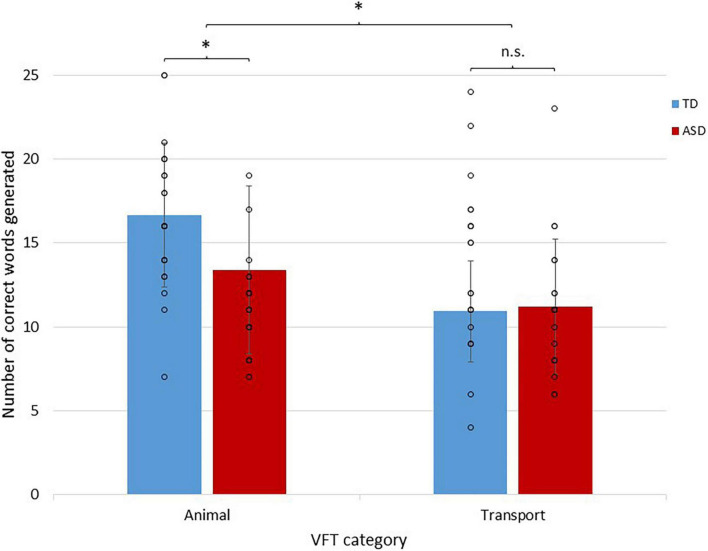
A bar chart showing semantic fluency performance in ASD and TD individuals during different semantic categories. Individual data points are represented by circles. Error bars represent ± 1 standard deviation of the mean. *p <0.05.

Regarding reading comprehension performance, a 2 × 3 mixed ANOVA showed a highly significant main effect of group [*F*_(1,40)_ = 7.58, *p* = 0.009] with a non-significant group by level interaction effect [*F*_(2,80)_ = 0.221, *p* = 0.802]. *Post hoc* independent *t*-tests for each difficulty level with Bonferroni corrections showed that ASD individuals performed poorer on high-level questions than TD individuals (*p* = 0.016; Table 2). Regarding semantic verbal fluency performance (Figure 3), a 2 × 2 mixed ANOVA showed a significant group by condition interaction effect [*F*_(1,42)_ = 4.90, *p* = 0.032] with a non-significant main effect of group [*F*_(1,42)_ = 2.04, *p* = 0.161]. *Post-hoc t*-tests with Bonferroni corrections showed that ASD individuals performed poorer in animal (*t*_42_ = 2.33, *p* = 0.024), but not transport (*t*_42_ = –0.265, *p* = 0.792), word generation. Regarding information processing efficiency, ASD individuals exhibited slower processing speed in CANTAB RTI five-choice mean reaction time (ASD mean = 473.87 ms, *S.D.* = 60.19 ms; TD mean = 430.24 ms, *S.D.* = 59.49 ms; *t*_42_ = 2.36, *p* = 0.023).

**TABLE 2:** Reading comprehension performance.

Parameters	Group				
	ASD	TD	*t*	*df*	*p*
**Reading comprehension total score**					
Main effect of group: *F*_(1, 40)_ = 7.58**, *p* = 0.009					
ART-C reading comprehension (low level) subscore (S.D.)	50.34 (36.84)	71.76 (34.83)	2.15	40	0.038
ART-C reading comprehension (medium level) subscore (S.D.)	61.39 (44.20)	88.35 (38.06)	2.30	40	0.027
ART-C reading comprehension (high level) subscore (S.D.)	49.52 (29.98)	68.78 (19.90)	2.95*	40	0.016

ART-C, Aligned Rank Transform Contrast.

ASD n = 19; TD n = 23; two data from the ASD group were missing.

*p < 0.017 (Bonferroni-corrected).

**p < 0.01 (Bonferroni-corrected).

### Functional near-infrared spectroscopy prefrontal activation during the semantic verbal fluency task

**FIGURE 4:**
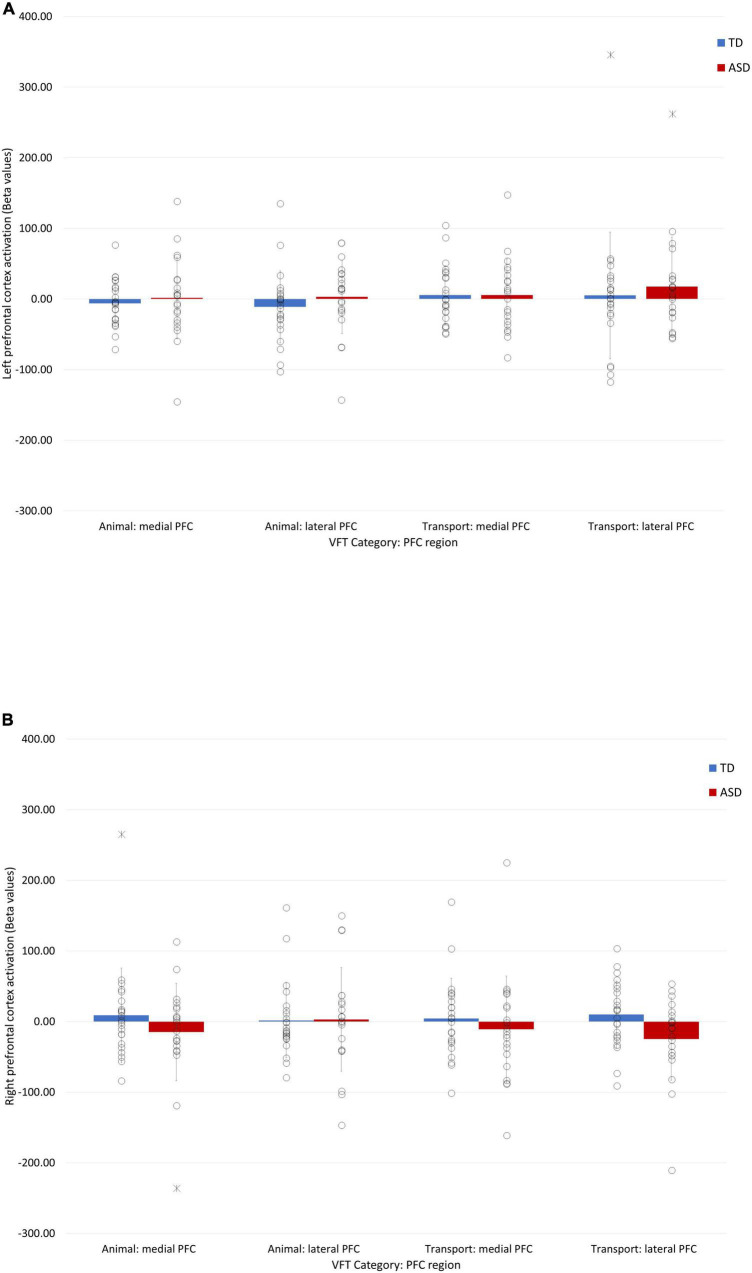
Bar charts showing the fNIRS prefrontal activation (beta values) during animal and transport word generation in the right **(A)** and left **(B)** hemispheres. Individual data points are represented by circles. Extreme outliers are represented by asterisks. Error bars represent ± 1 standard deviation of the mean.

Four extreme outliers (shown in Figure 4) were identified and removed from the analysis. A 4-way (Group × Condition × region × hemisphere) mixed ANOVA revealed a non-significant main effect of group (*p* = 0.67). All interaction effects were also non-significant (*p* > 0.06).

### Functional near-infrared spectroscopy prefrontal functional connectivity during the semantic verbal fluency task

Regarding intrahemispheric functional connectivity within the medial and lateral PFC (Table 3), a 4-way (Group × Condition × region × hemisphere) mixed ANOVA revealed a highly significant main effect of group [*F*_(1,42)_ = 11.36, *p* = 0.002] and a significant region × group interaction [*F*_(1,42)_ = 6.92, *p* = 0.012]. In both animal and transport word generation, ASD individuals exhibited a lower PFC functional connectivity in all subregions in both hemispheres, but only the lower intrahemispheric functional connectivity in the left medial PFC during animal (*t*_42_ = 2.92, *p* = 0.006) and transport (*t*_42_ = 3.13, *p* = 0.003) word generation survived Bonferroni corrections. Other interaction effects remained non-significant (*p*s > 0.085).

**TABLE 3:** Intrahemisphere within-region functional connectivity during verbal fluency task in ASD and TD individuals (Z-transformed coherence values).

	Left hemisphere					Right hemisphere				
	Group					Group				
	ASD	TD	*t*	*df*	*p*	ASD	*TD*	*t*	*df*	*p*
Main effect of group: *F*_(**1, 42)**_ = 11.36**. *p* = 0.002										
Group*condition*region*hemisphere: *F*_(**1, 42**)_ = 0.364, *p* = 0.550										
***Condition 1: Animal word generation***										
Medial	0.12 (0.12)	0.27 (0.22)	2.93**	42	0.006	0.17 (0.11)	0.26 (0.13)	2.65	42	0.011
Lateral	0.12 (0.10)	0.19 (0.12)	2.15	42	0.037	0.10 (0.09)	0.14 (0.07)	1.61	42	0.114
***Condition 2: Transport word generation***										
Medial	0.14 (0.12)	0.30 (0.21)	3.03**	42	0.004	0.12 (0.11)	0.22 (0.18)	2.27	42	0.029
Lateral	0.15 (0.11)	0.22 (0.14)	2.03	42	0.049	0.11 (0.09)	0.12 (0.07)	0.451	42	0.655

**p < 0.0063 (Bonferroni-corrected).

Regarding interhemispheric functional connectivity (Table 4), a 3-way (Group × Condition × region) mixed ANOVA revealed a highly significant main effect of group [*F*_(1,42)_ = 7.79. *p* = 0.008] and a significant region × group interaction [*F*_(1,42)_ = 4.35, *p* = 0.043]. ASD individuals exhibited lower interhemispheric functional connectivity in the lateral PFC during animal word generation (*t*_42_ = 2.73, *p* = 0.009), while lower interhemispheric medial PFC functional connectivity was observed in the transport word generation condition (*t*_42_ = 2.75, *p* = 0.008). Other interaction effects remained non-significant (*p*s > 0.076).

**TABLE 4:** Interhemispheric functional connectivity during verbal fluency task in ASD and TD individuals (Z-transformed connectivity index).

Prefrontal brain region	Group				
	ASD	TD	*t*	*df*	*p*
Main effect of group: *F*_(**1**, **42**)_ = 7.79**, *p* = 0.008					
Group*condition*region: *F*_(**1, 42**)_ = 4.35, *p* = 0.043					
***Condition 1: Animal word generation***					
Medial	0.18 (0.11)	0.27 (0.15)	2.37	42	0.023
Lateral	0.09 (0.08)	0.15 (0.08)	2.73**	42	0.009
***Condition 2: Transport word generation***					
Medial	0.16 (0.10)	0.28 (0.17)	2.75**	42	0.008
Lateral	0.11 (0.08)	0.15 (0.09)	1.54	42	0.13

**p < 0.0125 (Bonferroni-corrected).

### Brain-behavior relationships

**FIGURE 5:**
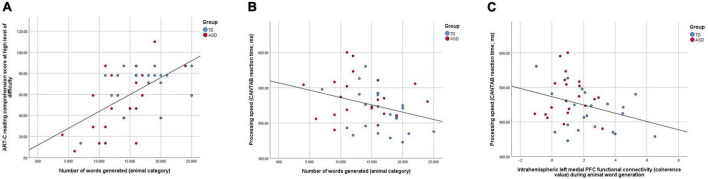
**(A)** A scatter plot showing a highly significant positive correlation between reading comprehension performance in the high level of difficulty and semantic verbal fluency performance in the animal category (*r*_42_ = 0.508, *p* < 0.001); **(B)** a scatter plot showing a significant negative correlation between semantic verbal fluency performance and processing speed (*r*_42_ = –0.312, *p* = 0.044); **(C)** a scatter plot showing a significant negative correlation between processing speed and left medial PFC functional connectivity during animal word generation (*r*_42_ = –0.319, *p* = 0.040).

The relationships between the strength of intrahemispheric functional connectivity in the left medial PFC and interhemispheric functional connectivity in the lateral PFC during animal word generation, processing speed, animal category verbal fluency performance and reading comprehension performance were examined. At the whole-group level, better animal word generation ability was found to be highly associated with better reading comprehension performance at a high level of difficulty (*r*_42_ = 0.508, *p* < 0.001; Figure 5A), which was also correlated with faster processing speed (*r*_42_ = –0.312, *p* = 0.044; Figure 5B). In turn, faster processing speed was significantly correlated with higher left medial PFC functional connectivity during animal word generation (*r*_42_ = –0.319, *p* = 0.040, Figure 5C). Subgroup analyses of the TD group revealed marginal significance in the correlation between reaction time and reading comprehension performance at a high level of difficulty (*r*_22_ = –0.42, *p* = 0.058) as well as between reaction time and the total number of animal words generated (*r*_22_ = 0.389, *p* = 0.067). For the ASD group, a higher total number of animal word generations was significantly associated with better reading comprehension performance at a high level of difficulty (*r*_20_ = 0.61, *p* = 0.006). Other correlations remained non-significant (*p*s > 0.1).

## Discussion

Our results showed that high-functioning ASD children performed markedly poorer in the reading comprehension test, accompanied by a significantly slower information processing speed and fewer correct words generated in only the living things category (i.e., animal) in the semantic verbal fluency test. Regarding prefrontal hemodynamics, while ASD individuals exhibited brain activation patterns comparable to those of their healthy counterparts, they exhibited global intrahemispheric and interhemispheric hypoconnectivity in the PFC during the VF task. At the whole-group level, lower left medial PFC functional connectivity was significantly correlated with a slower processing speed, provided that such a reduction in processing speed was also correlated with poor animal word generation performance, a parameter that was highly correlated with a poorer reading comprehension performance at a high level of difficulty. We will discuss the above results in the following paragraphs.

Consistent with previous findings (Brown et al., 2013), our ASD sample exhibited general reading comprehension deficits when compared to their TD counterparts. Importantly, within the ASD sample as well as at the whole-group level, the positive relationship between the number of words generated in the animal category and reading comprehension ability is highly significant, which is in line with a previous study that reveals a clear association between executive function and reading comprehension in people with autism (Micai et al., 2021). Given the VF task parameter reflects one’s vocabulary size, which has been suggested to be a parameter reflecting the vocabulary knowledge of an individual (Dong et al., 2020), it is reasonable to observe a direct relationship between VF task and reading comprehension performance as vocabulary knowledge has shown to be fundamental to the reading processes (Braze et al., 2007, 2016). Specifically, the *post hoc t*-tests with Bonferroni corrections revealed that ASD individuals exhibited significant impairment when comprehending longer passages (approximately 100–200 words) involving social context (i.e., conversations between two people) and abstract concepts (i.e., metaphorical descriptions for an object). Indeed, previous studies have revealed that school-age children with high-functioning ASD showed impaired ability to identify correct information from written text with more than one paragraph (Saldaña and Frith, 2007), and this impairment was more pronounced when they were asked to answer inferential questions (Myles and Simpson, 2002; Tirado and Saldana, 2016). In addition, regarding the reading comprehension task adopted in our study, the metaphorical nature of the task at medium- and high-level of complexity further challenged individuals with ASD, given these people have been consistently shown to encounter difficulties in figurative language processing (Vulchanova et al., 2015; Kalandadze et al., 2018). This may possibly complicate the interpretation of results because figurative language processing is beyond the scope of vocabulary knowledge and reading processes. Taken together, these findings suggest that components in the reading comprehension task other than task complexity *per se* could contribute to the observed reading comprehension deficits in ASD, which warrants further investigation.

Consistent with a local study studying verbal fluency ability in adolescents with ASD (Yeung et al., 2019) and a study from another East Asian country (Inokuchi and Kamio, 2013), we found category-dependent semantic verbal fluency impairment in children with high-functioning ASD. Importantly, we further revealed that poorer semantic verbal fluency performance was associated with poorer reading comprehension performance at a high level of difficulty and less efficient information processing. Taken together with our observation in reading comprehension performance in ASD discussed above, these findings suggest that selective reading comprehension impairment in ASD may be associated with deficits in information processing. In addition, our findings may help explain why semantic verbal fluency impairment was not observed in some studies, such as in Ehlen et al. (2020). In Ehlen et al. (2020), participants were allowed 120 s instead of 60 s to complete a verbal fluency task. This increase in time may have reduced demand on information processing, which might result in comparable performance between ASD and TD individuals. Differences in culture may also play a role in the category-specific impairment in VF in ASD, which warrants further investigation.

We further investigated the neurophysiological mechanism underlying the verbal fluency task in terms of prefrontal activation and functional connectivity using fNIRS and found that ASD individuals exhibited global intrahemispheric and interhemispheric hypoconnectivity in the PFC. In line with our hypothesis and previous neuroimaging studies (Anderson et al., 2011; Zhu et al., 2014; Lee et al., 2016; Guo et al., 2020), abnormalities in both intrahemispheric and interhemispheric functional connectivity are evident in ASD patients when compared to their healthy counterparts during semantic verbal fluency tasks. Specifically, *post-hoc*-tests showed that the intrahemispheric functional connectivity of the left medial PFC is lower in ASD during both animal and transport word generation. For interhemispheric functional connectivity, while the lateral PFC was hypoconnected during animal word generation, the medial PFC was hypoconnected during transport word generation in ASD. Previous studies have shown that the medial PFC is the key hub for learning and recalling learned associations between context, events and corresponding adaptive responses (Euston et al., 2012). In the context of semantic fluency, the left medial PFC facilitates the recall of words that are relevant to a particular given category (i.e., animal, transportation), which are considered learned associations. Because individuals with ASD were found to have hypoconnected left medial PFC, they may less efficiently retrieve relevant responses when semantic cues are given during tasks, resulting in slower responses and the production of fewer correct words (i.e., poor performance) within a limited time (i.e., 60 s). If we apply the same logic to contextualize the hypoconnected left medial PFC during transport word generation, we should also expect significantly poorer behavioral performance in ASD than in TD individuals. Interestingly, our results show a comparable number of correct transport words generated by ASD and TD individuals, which may be due to the following: For TD individuals, transport word generation is consistently found to be more difficult for local populations (Chan and Poon, 1999; Chan and Chen, 2004). Moreover, different types of transportation are commonly found to be a theme of restricted interests in many ASD individuals (Mancil and Pearl, 2008). Thus, high-functioning ASD individuals, who are often found to have intact, or even greater, vocabulary reserve than TD individuals (Kim and Lord, 2013), may be more knowledgeable in words relevant to transportation, which in turn masked the expected deficits in verbal fluency. Although we identified a significant negative correlation between functional connectivity in the left medial PFC during animal word generation and reaction time (i.e., indicator of information processing efficiency) at the whole group level, this correlation did not survive in the subgroup analysis. Non-significant correlations in the subgroups might be due to a reduction in power with fewer participants included in the correlation analysis. Future studies might consider acquiring an adequate sample size to explore the relationship between prefrontal functional connectivity and reaction time within ASD individuals to confirm our claim. In terms of prefrontal activation during the verbal fluency task, consistent with a previous fMRI study (Baxter et al., 2019), we found no significant differences in activation patterns between ASD and TD children during the verbal fluency task. This finding contradicts a previous fNIRS study conducted by Yeung et al. (2019), who recruited adolescents with ASD. Multiple studies have revealed a differential brain activation pattern in children compared to adults during VF tasks (Holland et al., 2001; Gaillard et al., 2003). More studies are needed to investigate the effect of age on prefrontal activation during verbal fluency tasks in ASD.

## Limitations

With a homogeneous group of patients and matched controls, this study provided important findings to aid our understanding of reading comprehension deficits in school-aged children with ASD. However, several limitations should be noted to ensure the careful interpretation of the results. First, although we attempted to achieve spatial consistency and ensure proper coverage of the medial and lateral prefrontal cortex across individuals by strictly adhering to a commonly performed procedure in many fNIRS studies, that is, the landmark identification approach according to the 10–10 (20) system (Huang et al., 2015; Yeung et al., 2019), the medial and lateral PFC ROIs may not exactly anatomically correspond to these two regions. However, high-quality structural MRI image recording might not be feasible for the currently recruited population, since children are well known to produce many motion artifacts that significantly impede image quality (Yuan et al., 2009); therefore, the spatial localization of targeted regions could not be improved by this method either. To improve the reliability in region-of-interest identification, a toolbox for deciding the NIRS probe arrangement for targeting a specific region of interest based on prerecorded MRI images, such as fNIRS Optodes’ Location Decider (fOLD, Zimeo Morais et al., 2018), could be used in future studies. Moreover, our sample only included high-functioning, right-handed ASD males, which limits the generalizability of the results. Given that previous studies have documented observable differences in behavioral VF performance between females and males (Burton et al., 2005; Sokołowski et al., 2020) and brain lateralization during VF tasks for people with different hand dominance (Gao et al., 2015), future studies might consider investigating how sex and handedness may contribute to differential PFC functional connectivity patterns during semantic VF tasks. In addition, verbal ASD subjects, regardless of their level of IQ, could be included for investigation, although this inclusion may also increase the heterogeneity of the sample, which could possibly contribute to other issues regarding results interpretation.

## Conclusion

This study aimed to investigate the neurophysiological mechanism underlying reading comprehension performance in children with high-functioning ASD aged 8–12 years. Twenty-one right-handed boys with high-functioning ASD and 23 age-, IQ-, education-, sex-, and handedness-matched TD individuals underwent a reading comprehension test, an information processing efficiency test, and a semantic verbal fluency test with concurrent fNIRS measurement. The results showed that high-functioning ASD children had impaired reading comprehension, with comprehension performance at a high level of difficulty showing a significant association with poorer performance in semantic VF, a task tapping core executive functioning components involved in reading comprehension. In addition, poorer semantic VF performance was associated with slower information processing speed, which was contributed by reduced left medial PFC functional connectivity. Our study has provided important implications for the neuropsychological and neurophysiological mechanisms underlying reading comprehension deficits in ASD.

## Data availability statement

The raw data supporting the conclusions of this article will be made available by the authors, without undue reservation.

## Ethics statement

The studies involving human participants were reviewed and approved by Human Subjects Ethics Sub-Committee of the Hong Kong Polytechnic University. Written informed consent to participate in this study was provided by the participants’ legal guardian/next of kin.

## Author contributions

YH, MC, and MY contributed to the conception and design of the study. MC organized the database and wrote the first draft of the manuscript. MC and M-CC performed the statistical analysis. MY, S-MW, DL, and YH contributed to the data interpretation. All authors contributed to manuscript revision, read, and approved the submitted version.

## References

[B1] American Psychological Association (2013). *Diagnostic and Statistical Manual of Mental Disorders: DSM-5*, 5th Edn. Washington, DC: American Psychiatric Publishing, Inc. 10.1176/appi.books.9780890425596

[B2] Amir H. Malka D.-K. Gil S. Rahav B.-G. Merav C. Kobi D. (2020). Cognitive enhancement of healthy older adults using hyperbaric oxygen: a randomized controlled trial. *Aging* 12:13740. 10.18632/aging.103571 32589613 PMC7377835

[B3] Anderson J. S. Druzgal T. J. Froehlich A. DuBray M. B. Lange N. Alexander A. L. (2011). Decreased interhemispheric functional connectivity in autism. *Cereb. Cortex* 21 1134–1146. 10.1093/cercor/bhq190 20943668 PMC3077433

[B4] Barker J. W. Aarabi A. Huppert T. J. (2013). Autoregressive model based algorithm for correcting motion and serially correlated errors in fNIRS. *Biomed. Optics Express* 4 1366–1379. 10.1364/BOE.4.001366 24009999 PMC3756568

[B5] Baxter L. C. Nespodzany A. Walsh M. Wood E. Smith C. J. Braden B. B. (2019). The influence of age and ASD on verbal fluency networks. *Res. Autism Spect. Disord.* 63 52–62. 10.1016/j.rasd.2019.03.002 32565886 PMC7304570

[B6] Beacher F. D. Radulescu E. Minati L. Baron-Cohen S. Lombardo M. V. Lai M.-C. (2012). Sex differences and autism: brain function during verbal fluency and mental rotation. *PLoS One* 7:e38355. 10.1371/journal.pone.0038355 22701630 PMC3373504

[B7] Braze D. Katz L. Magnuson J. S. Mencl W. E. Tabor W. Van Dyke J. A. (2016). Vocabulary does not complicate the simple view of reading. *Read. Writ.* 29 435–451. 10.1007/s11145-015-9608-6 26941478 PMC4761369

[B8] Braze D. Tabor W. Shankweiler D. P. Mencl W. E. (2007). Speaking up for vocabulary: Reading skill differences in young adults. *J. Learn. Disabil.* 40 226–243. 10.1177/00222194070400030401 17518215 PMC2847434

[B9] Brown H. M. Oram-Cardy J. Johnson A. (2013). A meta-analysis of the reading comprehension skills of individuals on the autism spectrum. *J. Autism Dev. Disord.* 43 932–955. 10.1007/s10803-012-1638-1 23054199

[B10] Burton L. A. Henninger D. Hafetz J. (2005). Gender differences in relations of mental rotation, verbal fluency, and SAT scores to finger length ratios as hormonal indexes. *Dev. Neuropsychol.* 28 493–505. 10.1207/s15326942dn2801_3 15992253

[B11] Butterfuss R. Kendeou P. (2018). The role of executive functions in reading comprehension. *Educ. Psychol. Rev.* 30 801–826. 10.1007/s10648-017-9422-6

[B12] Carmo J. C. Duarte E. Pinho S. Marques J. F. Filipe C. N. (2015). Verbal fluency as a function of time in autism spectrum disorder: an impairment of initiation processes? *J. Clin. Exp. Neuropsychol.* 37 710–721. 10.1080/13803395.2015.1062082 26207691

[B13] Carretti B. Borella E. Cornoldi C. De Beni R. (2009). Role of working memory in explaining the performance of individuals with specific reading comprehension difficulties: a meta-analysis. *Learn. Individ. Diff.* 19 246–251. 10.1016/j.lindif.2008.10.002

[B14] Carretti B. Cornoldi C. De Beni R. Romanò M. (2005). Updating in working memory: a comparison of good and poor comprehenders. *J. Exp. Child Psychol.* 91 45–66. 10.1016/j.jecp.2005.01.005 15814095

[B15] Chan A. S. Poon M. W. (1999). Performance of 7-to 95-year-old individuals in a Chinese version of the category fluency test. *J. Int. Neuropsychol. Soc.* 5 525–533. 10.1017/s135561779956606x 10561933

[B16] Chan M. M. Chan M.-C. Lai O. L.-H. Krishnamurthy K. Han Y. M. (2022). Abnormal prefrontal functional connectivity is associated with inflexible information processing in patients with Autism Spectrum Disorder (ASD): an fNIRS study. *Biomedicines* 10:1132. 10.3390/biomedicines10051132 35625869 PMC9139038

[B17] Chan R. C. Chen E. Y. (2004). Development of a Chinese verbal fluency test for the Hong Kong psychiatric setting. *Hong Kong J. Psychiatry* 14 8–12. 10.1016/S1569-1861(09)70024-5

[B18] Christopher M. E. Miyake A. Keenan J. M. Pennington B. DeFries J. C. Wadsworth S. J. (2012). Predicting word reading and comprehension with executive function and speed measures across development: a latent variable analysis. *J. Exp. Psychol.* 141:470. 10.1037/a0027375 22352396 PMC3360115

[B19] Constantino J. N. Gruber C. P. (2012). *Social Responsiveness Scale: SRS-2.* Torrance, CA: Western psychological services.

[B20] Cromley J. G. Azevedo R. (2007). Testing and refining the direct and inferential mediation model of reading comprehension. *J. Educ. Psychol.* 99:311. 10.1037/0022-0663.99.2.311

[B21] Demetriou E. A. Lampit A. Quintana D. S. Naismith S. L. Song Y. J. Pye J. E. (2018). Autism spectrum disorders: a meta-analysis of executive function. *Mol. Psychiatry* 23 1198–1204. 10.1038/mp.2017.75 28439105 PMC5984099

[B22] Dong Y. Tang Y. Chow B. W.-Y. Wang W. Dong W.-Y. (2020). Contribution of vocabulary knowledge to reading comprehension among Chinese students: a meta-analysis. *Front. Psychol.* 11:525369. 10.3389/fpsyg.2020.52536 33132948 PMC7561676

[B23] Ehlen F. Roepke S. Klostermann F. Baskow I. Geise P. Belica C. (2020). Small semantic networks in individuals with autism spectrum disorder without intellectual impairment: a verbal fluency approach. *J. Autism Dev. Disord.* 50 3967–3987. 10.1007/s10803-020-04457-9 32198662 PMC7560923

[B24] Elkin L. A. Kay M. Higgins J. J. Wobbrock J. O. (2021). “An aligned rank transform procedure for multifactor contrast tests,” in *The 34th Annual ACM Symposium on User Interface Software and Technology*, 10.1145/3472749.3474784 New York, NY.

[B25] Euston D. R. Gruber A. J. McNaughton B. L. (2012). The role of medial prefrontal cortex in memory and decision making. *Neuron* 76 1057–1070. 10.1016/j.neuron.2012.12.002 23259943 PMC3562704

[B26] Fray P. J. Robbins T. W. Sahakian B. J. (1996). Neuorpsychiatyric applications of CANTAB. *Int. J. Geriatr. Psychiatry* 11 329–336. 10.1002/(SICI)1099-1166(199604)11:4<329::AID-GPS453>3.0.CO;2-6

[B27] Gabig C. S. (2010). Phonological awareness and word recognition in reading by children with autism. *Commun. Disord. Q.* 31 67–85. 10.1177/1525740108328410

[B28] Gaillard W. D. Sachs B. C. Whitnah J. R. Ahmad Z. Balsamo L. M. Petrella J. R. (2003). Developmental aspects of language processing: fMRI of verbal fluency in children and adults. *Hum. Brain Mapp.* 18 176–185. 10.1002/hbm.10091 12599275 PMC6871939

[B29] Gao Q. Wang J. Yu C. Chen H. (2015). Effect of handedness on brain activity patterns and effective connectivity network during the semantic task of Chinese characters. *Sci. Rep.* 5:18262. 10.1038/srep18262 26666706 PMC4678893

[B30] García-Madruga J. A. Vila J. Gómez-Veiga I. Duque G. Elosúa M. R. (2014). Executive processes, reading comprehension and academic achievement in 3th grade primary students. *Learn. Individ. Diff.* 35 41–48. 10.1016/j.lindif.2014.07.013

[B31] Gough P. B. Tunmer W. E. (1986). Decoding, reading, and reading disability. *Remed. Spec. Educ.* 7 6–10. 10.1177/074193258600700104

[B32] Guo X. Duan X. Chen H. He C. Xiao J. Han S. (2020). Altered inter-and intrahemispheric functional connectivity dynamics in autistic children. *Hum. Brain Mapp.* 41 419–428. 10.1002/hbm.24812 31600014 PMC7268059

[B33] Han Y. M. Chan A. S. (2017). Disordered cortical connectivity underlies the executive function deficits in children with autism spectrum disorders. *Res. Dev. Disabil.* 61 19–31. 10.1016/j.ridd.2016.12.010 28042973

[B34] Han Y. M. Chan M.-C. Chan M. M. Yeung M. K. Chan A. S. (2022). Effects of working memory load on frontal connectivity in children with autism spectrum disorder: a fNIRS study. *Sci. Rep.* 12:1522. 10.1038/s41598-022-05432-3 35087126 PMC8795357

[B35] Hansen L. B. Morales J. Macizo P. Duñabeitia J. A. Saldaña D. Carreiras M. (2017). Reading comprehension and immersion schooling: evidence from component skills. *Dev. Sci.* 20:e12454. 10.1111/desc.12454 28032442

[B36] Holland S. K. Plante E. Byars A. W. Strawsburg R. H. Schmithorst V. J. Ball W. S. Jr. (2001). Normal fMRI brain activation patterns in children performing a verb generation task. *Neuroimage* 14 837–843. 10.1006/nimg.2001.0875 11554802

[B37] Hoover W. A. Gough P. B. (1990). The simple view of reading. *Read. Writ.* 2 127–160. 10.1007/BF00401799

[B38] Huang C.-J. Chou P.-H. Wei H.-L. Sun C.-W. (2015). Functional connectivity during phonemic and semantic verbal fluency test: a multichannel near infrared spectroscopy study. *IEEE J. Select. Top. Quant. Electron.* 22 43–48. 10.1109/JSTQE.2015.2503318

[B39] Inokuchi E. Kamio Y. (2013). Qualitative analyses of verbal fluency in adolescents and young adults with high-functioning autism spectrum disorder. *Res. Autism Spect. Disord.* 7 1403–1410. 10.1016/j.rasd.2013.08.010

[B40] Jones C. R. Happé F. Golden H. Marsden A. J. Tregay J. Simonoff E. (2009). Reading and arithmetic in adolescents with autism spectrum disorders: peaks and dips in attainment. *Neuropsychology* 23 718. 10.1037/a0016360 19899830

[B41] Kalandadze T. Norbury C. Nærland T. Næss K.-A. B. (2018). Figurative language comprehension in individuals with autism spectrum disorder: a meta-analytic review. *Autism* 22 99–117. 10.1177/1362361316668652 27899711 PMC5843023

[B42] Kendeou P. McMaster K. L. Christ T. J. (2016). Reading comprehension: core components and processes. *Behav. Brain Sci.* 3 62–69. 10.1177/2372732215624707

[B43] Kenworthy L. Wallace G. L. Birn R. Milleville S. C. Case L. K. Bandettini P. A. (2013). Aberrant neural mediation of verbal fluency in autism spectrum disorders. *Brain Cogn.* 83, 218–226. 10.1016/j.bandc.2013.08.003 24056237 PMC3829693

[B44] Kim S. H. Lord C. (2013). “The behavioral manifestations of autism spectrum disorders,” in *The neuroscience of autism spectrum disorders*, eds Buxbaum J. Hof P. R. (White Plains, NY: Center for autism and the developing brain). 10.1016/B978-0-12-391924-3.00002-8

[B45] Kim S. H. Paul R. Tager-Flusberg H. Lord C. (2014). “Language and communication in autism,” in *Handbook of autism and pervasive developmental disorders*, 4th Edn, eds Volkmar F. R. Rogers S. J. Paul R. Pelphrey K. A. (Hoboken, NJ: John Wiley & Sons, Inc).

[B46] Kim Y.-S. G. (2020). Toward integrative reading science: the direct and indirect effects model of reading. *J. Learn. Disabil.* 53 469–491. 10.1177/0022219420908239 32125226

[B47] King J. B. Prigge M. B. King C. K. Morgan J. Weathersby F. Fox J. C. (2019). Generalizability and reproducibility of functional connectivity in autism. *Mol. Autism* 10 1–23. 10.1186/s13229-019-0273-5 31285817 PMC6591952

[B48] Koshino H. Carpenter P. A. Minshew N. J. Cherkassky V. L. Keller T. A. Just M. A. (2005). Functional connectivity in an fMRI working memory task in high-functioning autism. *Neuroimage* 24 810–821. 10.1016/j.neuroimage.2004.09.028 15652316

[B49] Lai M.-C. Lombardo M. V. Ruigrok A. N. Chakrabarti B. Wheelwright S. J. Auyeung B. (2012). Cognition in males and females with autism: similarities and differences. *PLoS One* 7:e47198. 10.1371/journal.pone.0047198 23094036 PMC3474800

[B50] Lee J. M. Kyeong S. Kim E. Cheon K.-A. (2016). Abnormalities of inter-and intra-hemispheric functional connectivity in autism spectrum disorders: a study using the autism brain imaging data exchange database. *Front. Neurosci.* 10:191. 10.3389/fnins.2016.00191 27199653 PMC4853413

[B51] Lezak M. D. Howieson D. B. Loring D. W. Fischer J. S. (2004). *Neuropsychological assessment.* Oxford: Oxford University Press.

[B52] Lord C. Rutter M. Le Couteur A. (1994). Autism Diagnostic Interview-Revised: a revised version of a diagnostic interview for caregivers of individuals with possible pervasive developmental disorders. *J. Autism Dev. Disord.* 24 659–685. 10.1007/BF02172145 7814313

[B53] Mancil G. R. Pearl C. E. (2008). Restricted interests as motivators: improving academic engagement and outcomes of children on the autism spectrum. *Teach. Except. Child. Plus* 4:n6.

[B54] Martin J. D. Shipstead Z. Harrison T. L. Redick T. S. Bunting M. Engle R. W. (2020). The role of maintenance and disengagement in predicting reading comprehension and vocabulary learning. *J. Exp. Psychol.* 46:140. 10.1037/xlm0000705 31169403

[B55] Micai M. Vulchanova M. Saldaña D. (2021). Reading goals and executive function in autism: an eye-tracking study. *Autism Res.* 14 1007–1024. 10.1002/aur.2447 33278333

[B56] Minshew N. J. Williams D. L. McFadden K. (2008). “Information processing, neural connectivity, and neuronal organization,” in *Autism*, ed. Zimmerman A. W. (Berlin: Springer), 381–405. 10.1007/978-1-60327-489-0_18

[B57] Miyake A. Friedman N. P. Emerson M. J. Witzki A. H. Howerter A. Wager T. D. (2000). The unity and diversity of executive functions and their contributions to complex “frontal lobe” tasks: a latent variable analysis. *Cogn. Psychol.* 41 49–100. 10.1006/cogp.1999.0734 10945922

[B58] Moje E. B. Stockdill D. Kim K. Kim H.-J. (2011). “The role of text in disciplinary learning,” *Handbook of reading research*, eds Kamil M. Pearson P. D. Mosenthal P. Afflerbach P. Moje E. B. (Mahwah, NJ: Erlbaum/Taylor & Francis).

[B59] Myles B. S. Simpson R. L. (2002). Asperger syndrome: an overview of characteristics. *Focus Autism Other Dev. Disabil.* 17 132–137. 10.1177/10883576020170030201

[B60] Neubauer A. C. Fink A. (2003). Fluid intelligence and neural efficiency: effects of task complexity and sex. *Pers. Individ. Diff.* 35 811–827. 10.1016/S0191-8869(02)00285-4

[B61] Oldfield R. C. (1971). The assessment and analysis of handedness: the Edinburgh inventory. *Neuropsychologia* 9 97–113. 10.1016/0028-3932(71)90067-4 5146491

[B62] Ota T. Iida J. Okazaki K. Ishida R. Takahashi M. Okamura K. (2020). Delayed prefrontal hemodynamic response associated with suicide risk in autism spectrum disorder. *Psychiatry Res.* 289:112971. 10.1016/j.psychres.2020.112971 32408192

[B63] Paschoal A. M. da Silva P. H. R. Rondinoni C. Arrigo I. V. Paiva F. F. Leoni R. F. (2021). Semantic verbal fluency brain network: delineating a physiological basis for the functional hubs using dual-echo ASL and graph theory approach. *J. Neural Engineer.* 18:046089. 10.1088/1741-2552/ac0864 34087805

[B64] Penny W. D. Friston K. J. Ashburner J. T. Kiebel S. J. Nichols T. E. (2011). *Statistical parametric mapping: the analysis of functional brain images.* Amsterdam: Elsevier.

[B65] Pretorius E. J. (2002). Reading ability and academic performance in South Africa: Are we fiddling while Rome is burning? *Lang. Matt.* 33 169–196. 10.1080/10228190208566183

[B66] Reverberi C. Laiacona M. Capitani E. (2006). Qualitative features of semantic fluency performance in mesial and lateral frontal patients. *Neuropsychologia* 44 469–478. 10.1016/j.neuropsychologia.2005.05.011 16005031

[B67] Robinson G. Shallice T. Bozzali M. Cipolotti L. (2012). The differing roles of the frontal cortex in fluency tests. *Brain* 135 2202–2214. 10.1093/brain/aws142 22669082 PMC3381725

[B68] Sachse M. Schlitt S. Hainz D. Ciaramidaro A. Schirman S. Walter H. (2013). Executive and visuo-motor function in adolescents and adults with autism spectrum disorder. *J. Autism Dev. Disord.* 43 1222–1235. 10.1007/s10803-012-1668-8 23011252

[B69] Saldaña D. Frith U. (2007). Do readers with autism make bridging inferences from world knowledge? *J. Exp. Child Psychol.* 96 310–319. 10.1016/j.jecp.2006.11.002 17196973

[B70] Santosa H. Aarabi A. Perlman S. B. Huppert T. (2017). Characterization and correction of the false-discovery rates in resting state connectivity using functional near-infrared spectroscopy. *J. Biomed. Optics* 22:055002. 10.1117/1.JBO.22.5.055002 28492852 PMC5424771

[B71] Santosa H. Zhai X. Fishburn F. Huppert T. (2018). The NIRS brain AnalyzIR toolbox. *Algorithms* 11:73. 10.3390/a11050073 PMC11218834 38957522

[B72] Savage R. Cornish K. Manly T. Hollis C. (2006). Cognitive processes in children’s reading and attention: the role of working memory, divided attention, and response inhibition. *Br. J. Psychol.* 97 365–385. 10.1348/000712605X81370 16848949

[B73] Shevlyakov G. Smirnov P. (2011). Robust estimation of the correlation coefficient: an attempt of survey. *Austrian J. Stat.* 40 147–156.

[B74] Silva P. H. R. D. Secchinato K. F. Rondinoni C. Leoni R. F. (2020). Brain structural–functional connectivity relationship underlying the information processing speed. *Brain Connect.* 10 143–154. 10.1089/brain.2019.0726 32183565

[B75] Sokołowski A. Tyburski E. Sołtys A. Karabanowicz E. (2020). Sex differences in verbal fluency among young adults. *Adv. Cogn. Psychol.* 16:92. 10.5709/acp-0288-1 32607136 PMC7311951

[B76] Swanson H. Howard C. Saez L. (2006). Components of working memory that are related to poor reading comprehension and word recognition performance in less skilled readers. *J. Learn. Disabil.* 39 252–269. 10.1177/00222194060390030501 16724796

[B77] Tirado M. J. Saldana D. (2016). Readers with autism can produce inferences, but they cannot answer inferential questions. *J. Autism Dev. Disord.* 46 1025–1037. 10.1007/s10803-015-2648-6 26547920

[B78] Venker C. E. Mathée J. Neumann D. Edwards J. Saffran J. Ellis Weismer S. (2021). Competing perceptual salience in a visual word recognition task differentially affects children with and without autism spectrum disorder. *Autism Res.* 14 1147–1162. 10.1002/aur.2457 33372400 PMC8192461

[B79] Vilenius-Tuohimaa P. M. Aunola K. Nurmi J. E. (2008). The association between mathematical word problems and reading comprehension. *Educ. Psychol.* 28 409–426. 10.1080/01443410701708228

[B80] Vulchanova M. Saldaña D. Chahboun S. Vulchanov V. (2015). Figurative language processing in atypical populations: the ASD perspective. *Front. Hum. Neurosci.* 9:24. 10.3389/fnhum.2015.00024 25741261 PMC4330886

[B81] Wagner S. Sebastian A. Lieb K. Tüscher O. Tadić A. (2014). A coordinate-based ALE functional MRI meta-analysis of brain activation during verbal fluency tasks in healthy control subjects. *BMC Neurosci.* 15:19. 10.1186/1471-2202-15-19 24456150 PMC3903437

[B82] Wechsler D. (2010). *Wechsler adult intelligence scale.* London: Pearson.

[B83] Woolley G. (2011). *Reading comprehension.* Berlin: Springer, 15–34. 10.1007/978-94-007-1174-7_2

[B84] Wu C.-Y. Ho M.-H. R. Chen S.-H. A. (2012). A meta-analysis of fMRI studies on Chinese orthographic, phonological, and semantic processing. *Neuroimage* 63 381–391. 10.1016/j.neuroimage.2012.06.047 22759996

[B85] Yeung M. K. Lee T. L. Chan A. S. (2019). Frontal lobe dysfunction underlies the differential word retrieval impairment in adolescents with high-functioning autism. *Autism Res.* 12 600–613. 10.1002/aur.2082 30758144

[B86] Yeung M. K. Lin J. (2021). Probing depression, schizophrenia, and other psychiatric disorders using fNIRS and the verbal fluency test: a systematic review and meta-analysis. *J. Psychiatr. Res.* 140 416–435. 10.1016/j.jpsychires.2021.06.015 34146793

[B87] Yuan W. Altaye M. Ret J. Schmithorst V. Byars A. W. Plante E. (2009). Quantification of head motion in children during various fMRI language tasks. *Hum. Brain Mapp.* 30 1481–1489. 10.1002/hbm.20616 18636549 PMC2763570

[B88] Zhu H. Fan Y. Guo H. Huang D. He S. (2014). Reduced interhemispheric functional connectivity of children with autism spectrum disorder: evidence from functional near infrared spectroscopy studies. *Biomed. Optics Express* 5 1262–1274. 10.1364/BOE.5.001262 24761305 PMC3985986

[B89] Zimeo Morais G. A. Balardin J. B. Sato J. R. (2018). fNIRS Optodes’ Location Decider (fOLD): a toolbox for probe arrangement guided by brain regions-of-interest. *Sci. Rep.* 8:3341. 10.1038/s41598-018-21716-z 29463928 PMC5820343

